# Conservation tillage dominates pore formation freeze thaw cycles alleviate compaction in northeast China black soil

**DOI:** 10.1016/j.isci.2026.116726

**Published:** 2026-07-14

**Authors:** Qinglin Li, Renjie Hou, Zhaoqiang Zhou, Qiang Fu, Tianxiao Li

**Affiliations:** 1 College of Hydraulic Science and Engineering, Northeast Agricultural University, Harbin, Heilongjiang 150030, P.R. China; 2 State Key Laboratory of Soil and Sustainable Agriculture, Institute of Soil Science, Chinese Academy of Sciences, Nanjing 210008, P.R. China; 3 Heilongjiang Provincial Key Laboratory of Water Resources and Water Conservancy Engineering in Cold Region, Northeast Agricultural University, Harbin, Heilongjiang 150030, P.R. China; 4 International Cooperation Joint Laboratory of Health in Cold Region Black Soil Habitat of the Ministry of Education, Northeast Agricultural University, Harbin, Heilongjiang 150030, P.R. China

**Keywords:** conservation tillage, mollisols, rotary tillage, soil pore structure, x-ray computed tomography

## Abstract

Conservation tillage (CT) enhances soil structure, but its interaction with freeze-thaw cycles on micropores remains unclear. This field study compared CT and rotary tillage (RT) by using X-ray computer tomography to analyze soil pore characteristics and physical properties. CT promoted large pores (>500 μm) forming stable preferential flow paths, while RT mainly developed medium-sized pores (200–400 μm). Freeze-thaw cycles increased >300 μm porosity by 5%, particularly in the 20–40 cm layer, an effect influenced by bulk density and freezing duration. Soil pore parameters contributed 35% and 48% to soil nutrients in the 0–20 cm and 20–40 cm layers, respectively. Overall, tillage dominates soil porosity formation, and freeze-thaw cycles play a supplementary role in alleviating compaction. These findings provide practical guidelines for integrating CT with natural freeze-thaw processes to sustain black soil health in cold regions.

## Highlights

• Mechanical tillage creates stratified soil hardness
• Conservation tillage promotes large macropores over 500 μm in the topsoil
• Tillage primarily alters soil porosity, freeze-thaw cycles secondarily
• Conservation tillage promotes the formation of stable preferential flow pathways

## Graphical abstract

**Figure:**
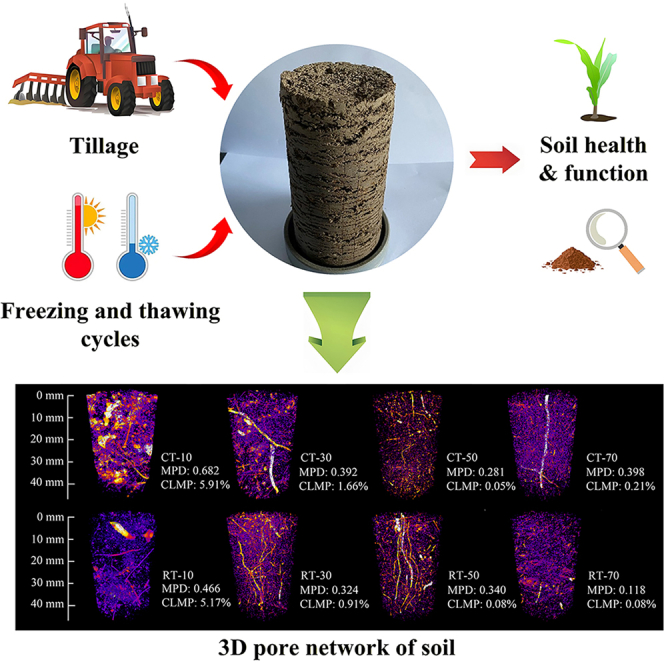

## Teaser

Earth-surface processes; soil science; agricultural science

## Introduction

The black soil region in northeast China covers an area of 1.2486 million km^2^ and serves as an important grain production base in China.^1^ In recent years, to enhance the efficiency of agricultural activities, increasing mechanical load and more frequent field rail transportation have damaged the soil structure in some areas,^2^ resulting in reduced water and nutrient storage capacity of rhizosphere soil, poor soil pore connectivity, and weakened soil water permeability.^3^^,^^4^^,^^5^ Given the “hardening” condition of black soil, it is imperative to modify traditional farming practices and establish a robust surface layer structure. Such measures are essential for maintaining the quality of cultivated land in black soil regions while promoting its protection, utilization, and fostering scientific and technological innovation.

Tillage is recognized as an effective method for changing soil pore structure.^6^ An important point is that plant residues resulting from conservation tillage can enhance the soil organic carbon content (SOC).^7^ As carbon accumulates over time, it mitigates the risk of soil compaction and facilitates improved connectivity within the topsoil.^8^^,^^9^ In addition, with an increasing duration of conservation tillage practices, it is easier to form a stable preferential flow path driven by biological pores, which promotes faster vertical transport of soil solution.^10^ However, some studies have indicated that there are no significant differences in pore characteristic parameters between conservation tillage and traditional tillage treatments.^11^ Based on these findings, it is evident that while conservation tillage holds considerable potential to alleviate soil compaction, its impact on soil structure may vary depending on specific local conditions.

In addition to the biological pores formed during plant growth, the pore structure of soil is also composed of biological pore channels generated by soil animal activities and abiotic pores formed by the mutual extrusion of aggregates caused by the freeze-thaw cycles (FTCs) and dry-wet cycles.^12^^,^^13^ During the study of the physical properties of subsoil, FTCs were found to significantly reduce soil bulk density (BD), increase soil porosity, and enhance soil saturated water conductivity.^14^ Through laboratory simulations of freeze-thaw conditions, it was established that the water retention effect associated with viscous layers formed during these cycles is a primary factor contributing to soil erosion.^15^ FTCs were observed to lead to an increase in elongated macropores within the soil aggregates of alpine meadows.^16^ As the number of FTCs increases, total porosity, long-hole porosity, and porosity for equivalent pore sizes greater than 100 μm are significantly enhanced; moreover, these cycles appear to weaken the stability of soil particles.^17^ The aforementioned research findings underscore that the impact of FTCs on abiotic pores within soils is considerable and warrants attention.

The pore structure of agricultural soil is often likened to a “black box”. Under the influence of external factors, the pore structure of soil has strong heterogeneity.^18^ However, with the rapid development of non-destructive detection of pore structure and computer simulation technology, pore imaging technology represented by X-ray computer tomography scanning technology makes soil structure and process more “transparent”.^19^ This technology not only captures the micro-pore structure of soil^20^ but also enables quantification of root configurations within the soil matrix^21^ and simulates water transport processes in soils.^22^ Consequently, X-ray computed tomography serves as a powerful tool for the quantitative analysis and visualization of soil pore structures.

Based on the compaction status of black soil, this study selected a test site for tillage return to field in 2022, located in a typical black soil area of northeast China, to explore the effects of two tillage methods (conservation tillage and rotary tillage) and of FTCs on basic physical properties and pore structure in different layers of typical black soil (0–20 cm, 20–40 cm, 40–60 cm, 60–80 cm). This study puts forward the following two hypotheses: (1) compared with rotary tillage, conservation tillage can enhance the surface soil’s water-conducting capacity and (2) the combination of FTCs and conservation tillage contributes to alleviating soil compaction. This research aims to provide a theoretical basis and technical support for the protection and sustainable utilization of land with black soil as well as rational cultivation practices in the typical black soil region of northeast China.

## Results

### Characteristics of soil temperature change under different tillage methods

**Figure 1:**
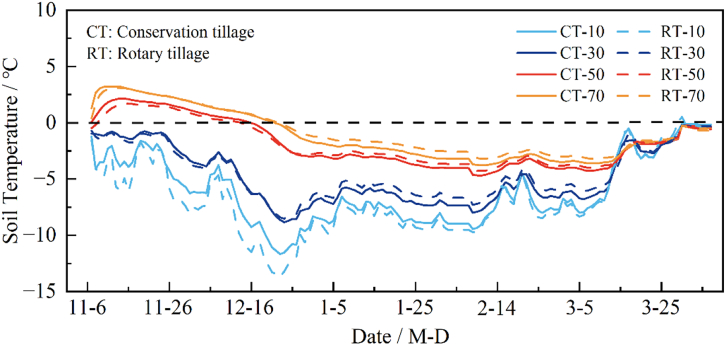
Temperature changes in soil under different tillage treatments

Temperature changes in different soil layers under conservation tillage and rotary tillage are shown in Figure 1. Affected by temperature fluctuations, the monitoring period revealed that the temperature variation range across different soil layers under the two treatments spanned from −11.67°C to 3.21°C. With the exception of the 0–20 cm soil layer, all other soil layers experienced a complete FTC. The overall soil temperature exhibited a trend of initially decreasing followed by an increase due to temperature fluctuations. Conservation tillage treatment revealed that the average temperature of the 0–20 cm soil layer was the lowest, recorded at −6.09°C. As depth increased, the average temperature of each soil layer increased successively, rising to −4.75°C, −1.89°C, and −1.11°C respectively. Moreover, both the 0–20 cm and the 20–40 cm soil layers reached their minimum temperatures of −11.67°C and −8.64°C, respectively, on December 23rd, but the lower layers (40–60 cm and 60–80 cm) experienced a delay in reaching their respective minimum temperatures of −4.69°C and −3.79°C on February 15th. The freezing duration of the bottom soil layer was about two-thirds of the surface soil layer, indicating a notable lag in both freezing and melting processes. When comparing temperature variations between conservation tillage treatment and rotary tillage treatment, it was observed that only within the 0–20 cm soil layer did conservation tillage exhibit significantly higher temperatures than rotary tillage—an increase averaging just −0.64°C was noted here alone. In addition, compared with the surface soil layer, the duration of soil freezing (DSF; taking 0°C as the threshold, it is defined as the duration during which the soil temperature decreases from 0°C to below 0°C and subsequently rises back to 0°C) in the bottom soil layer was shorter. In the 60–80 cm soil layer, the freezing duration under conservation tillage and rotary tillage treatment was only 108 and 107 days, which was much smaller than the 152 days observed in the 0–40 cm soil layer.

### Effects of tillage methods and freeze-thaw cycles on soil physical properties

**Figure 2:**
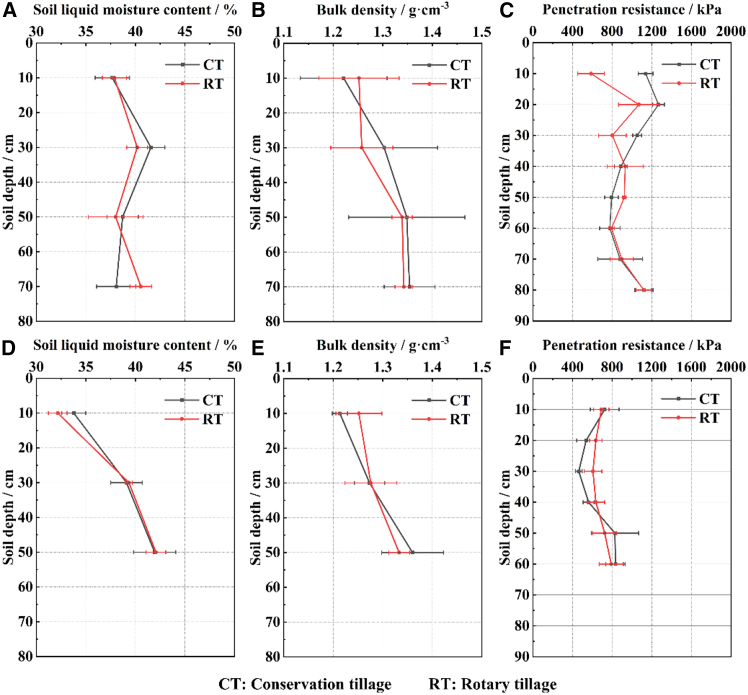
Changes in soil liquid moisture content, bulk density, and penetration resistance before and after freeze-thaw cycling (A) Soil liquid moisture content before freeze-thaw cycling. Data are represented as mean ± SEM. (B) Bulk density before freeze-thaw cycling. Data are represented as mean ± SEM. (C) Penetration resistance before freeze-thaw cycling. Data are represented as mean ± SEM. (D) Soil liquid moisture content after freeze-thaw cycling. Data are represented as mean ± SEM. (E) Bulk density after freeze-thaw cycling. Data are represented as mean ± SEM. (F) Penetration resistance after freeze-thaw cycling. Data are represented as mean ± SEM.

The results presented in Figure 2 indicate significant differences in the vertical distribution of soil liquid moisture content (SMC), soil BD, and penetration resistance (PR) across various tillage treatments. For the 0–20 cm soil layer, the SMC under the two treatments was similar, and the soil BD and soil PR under conservation tillage treatment were smaller and larger, repectively. As depth increased, the maximum SMC for the 20–40 cm layer under conservation tillage reached 41.58 cm^3^ cm^−3^, while that under rotary tillage treatment recorded a peak value of 40.55 cm^3^ cm^−3^ at depths of 60–80 cm. Furthermore, both treatments demonstrated an increasing trend in BD with greater depth. At a depth of 60 cm, changes in soil BD and PR followed similar patterns for both treatments. Following thawing events, water loss and infiltration were observed within the topsoil (0–20 cm), resulting in decreases in liquid water content by 3.97 cm^3^ cm^−3^ for conservation tillage and by 5.72 cm^3^ cm^−3^ for rotary tillage. As depth continued to increase, there was an upward trend in SMC; notably, soils within the 60–80 cm layer approached saturation levels that precluded further sampling analysis. Additionally, it is important to highlight that FTCs contributed to reductions in both BD and PR within the studied soils.

The variations in soil saturated water conductivity are presented in Table 1. The maximum saturated water conductivity was 9.78 × 10^−2^ mm min^−1^ under conservation tillage treatment of the 0–20 cm soil layer. Following FTCs, the saturated water conductivity of soil increased to 1.17 × 10^−1^ mm min^−1^. The saturated water conductivity of the 20–40 cm soil layer was higher than that of conservation tillage treatment, and the value decreased under the influence of FTCs. In the 40–60 cm soil layer, soil saturated hydraulic conductivity after FTC was better than that before FTC. Overall, an increasing soil depth correlated with a decreasing trend in soil saturated water conductivity.

**Table 1:** Soil saturated hydraulic conductivity of each layer under different treatments (mm·min^−1^)

Stage	Treatment	10 cm	30 cm	50 cm	70 cm
Before freezing	CT	9.78 × 10^−2^ Ab	4.23 × 10^−4^ Ba	2.78 × 10^−3^ Aa	3.00 × 10^−4^ B
	RT	6.34 × 10^−2^ Bb	3.09 × 10^−3^ Aa	1.17 × 10^−3^ Ab	2.46 × 10^−3^ A
After melting	CT	1.17 × 10^−1^ Aa	3.46 × 10^−4^ Ba	3.68 × 10^−3^ Aa	–
	RT	1.00 × 10^−1^ Aa	2.74 × 10^−3^ Aa	4.58 × 10^−3^ Aa	–

To facilitate comparison, 1 × 10^−2^ mm min^−1^ = 0.06 cm h^−1^.

CT, conservation tillage; RT, rotary tillage.

Capital letters indicate significant differences between the two tillage methods, whereas lowercase letters denote significant differences within the same treatment before freezing and after thawing.

### Pore structure characteristics of black soil under different tillage methods

**Figure 3:**
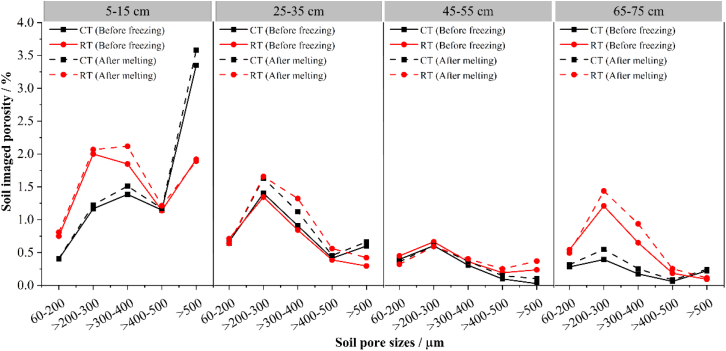
Distribution of soil pore sizes in various soil layers under different tillage modes (A) Distribution of soil pore sizes in 0–15 cm. CT represents conservation tillage, and RT represents rotary tillage. The distribution of pore sizes was classified based on the research conducted by Ding et al. (2023).^11^ (B) Distribution of soil pore sizes in 25–35 cm. (C) Distribution of soil pore sizes in 45–55 cm. (D) Distribution of soil pore sizes in 65–75 cm.

For the conservation tillage treatment, pores larger than 500 μm in the 0–20 cm soil layer accounted for approximately 45% of the total imaged porosity (Figure 3). In contrast, pores measuring between 200 and 300 μm in other soil layers represented the largest proportion, approximately 35% of the total imaged porosity. For rotary tillage treatment, although presence of pores >500 μm in the 0–20 cm soil layer was also relatively high, from the overall change in different soil layers, pores of 200–400 μm occupied the vast majority of the total imaged porosity (Figure 3). Furthermore, regardless of the tillage method, there was a notable decrease in the proportion of pores exceeding 300 μm as soil depth increased. Conversely, only a slight increase was observed in the proportion of pores ranging from 60 to 300 μm when comparing surface soils with sub-surface soils.

Under the influence of FTCs, an increase in soil imaged porosity was noted across all layers for both tillage methods. Notably, this increment was most pronounced within the 20–40 cm soil layer; specifically, conservation and rotary tillage treatments resulted in increases of 0.52% and 1.03%, respectively. A more detailed examination of changes in soil pore characteristics under varying aperture conditions revealed that within the topmost (0–20 cm) layer, it was primarily those with sizes between 300 and 400 μm that exhibited significant increases for both treatments; conversely, within the deeper (40–60 cm) layer, it was again those larger than 500 μm that showed marked growth.

**Figure 4:**
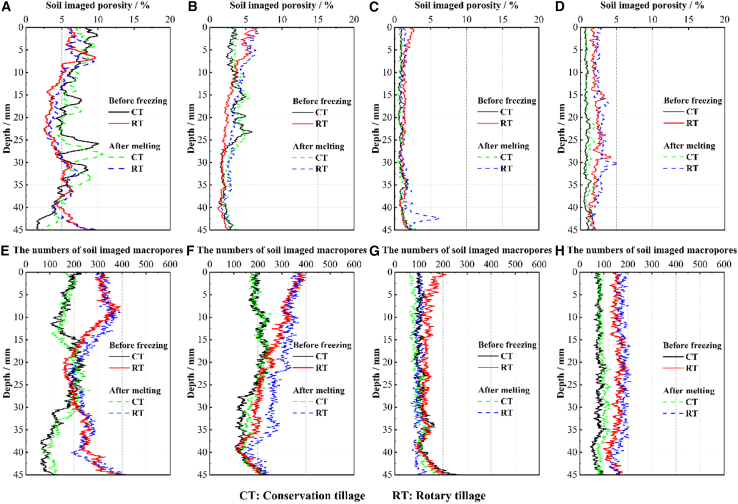
The variation of soil imaged porosity and the numbers of soil imaged macropores with depth (A) Variation of imaged porosity with depth in the 5–15 cm soil layer. The height in each sample from the ROI is about 45 mm. (B) Variation of imaged porosity with depth in the 25–35 cm soil layer. (C) Variation of imaged porosity with depth in the 45–55 cm soil layer. (D) Variation of imaged porosity with depth in the 65–75 cm soil layer. (E) Variation of the number of imaged macropores with depth in the 5–15 cm soil layer. (F) Variation of the number of imaged macropores with depth in the 25–35 cm soil layer. (G) Variation of the number of imaged macropores with depth in the 45–55 cm soil layer. (H) Variation of the number of imaged macropores with depth in the 65–75 cm soil layer.

Before FTCs, for the 0–20 cm soil layer, conservation tillage treatment yielded threshold values for imaged porosity and pore number ranging from 1.50% to 10.08% and between 61 and 248, respectively. In contrast, rotary tillage treatment resulted in threshold values for imaged porosity and pore number spanning from 2.51% to 9.68% and between 151 and 424, respectively. Under both tillage treatments, the maximum value of imaged porosity dropped below 3%, and the threshold value of soil pore number decreased. This observation suggests a transition from a mixture of large and small pores at shallower depths to predominantly regular small pores as one moves deeper into the profile. Following FTCs, with the exception of the 60–80 cm soil layer, there was a slight increase in both imaged porosity and pore number across soils subjected to either tillage method; however, this effect was significantly less pronounced compared with that induced by different tillage measures. Furthermore, Figure 4A illustrates that under conservation tillage treatment, the peak value of soil imaged porosity reached up to 25.86 mm before FTCs but increased to approximately 28.14 mm post-cycles; notably, despite these changes being similar in waveform pattern over time intervals examined, they indicate that thaw collapse occurred within the topsoil (0–20 cm) under conservation tillage conditions (Figure 4).

**Figure 5:**
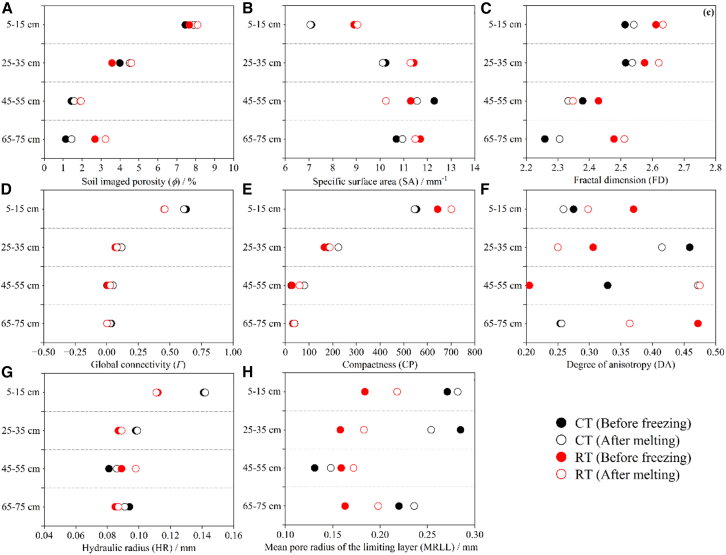
Soil pore characteristic parameters based on CT images (A) Changes in soil imaged porosity before and after freeze-thaw cycles under different tillage methods. (B) Changes in specific surface area before and after freeze-thaw cycles under different tillage methods. (C) Changes in fractal dimension before and after freeze-thaw cycles under different tillage methods. (D) Changes in global connectivity before and after freeze-thaw cycles under different tillage methods. (E) Changes in compactness before and after freeze-thaw cycles under different tillage methods. (F) Changes in degree of anisotropy before and after freeze-thaw cycles under different tillage methods. (G) Changes in hydraulic radius before and after freeze-thaw cycles under different tillage methods. (H) Changes in mean pore radius of the limiting layer before and after freeze-thaw cycles under different tillage methods.

Figure 5 recorded pore characteristic parameters under different tillage methods and across various soil layers, including soil imaged porosity, pore specific surface area (SA), fractal dimension (FD), global connectivity, tightness, anisotropy, hydraulic radius (HR), and average pore radius of the maximum confining layer. On the whole, in the surface soil (0–20 cm), the imaged porosity for rotary tillage treatment was slightly higher than that for conservation tillage soil by 0.2%, the specific SA of pores increased by 1.8 mm^−1^, the FD increased by 0.1, the global connectivity decreased by 0.17, the HR decreased by 0.03 mm, and the pore radius of the confining layer decreased by 0.09 mm. These results indicated that while rotary tillage promotes a better and more complex soil structure, its capacity for water and air transport is inferior to that observed with conservation tillage. However, with the increase of soil depth (40–80 cm), the global connectivity, tightness, and HR of soil decreased sharply, indicating that the pores of the underlying soil were independent of each other, and the connectivity became worse, which was not conducive to the transmission and exchange of soil water and air. Furthermore, Figure 5 illustrates that except for imaged porosity values influenced by FTCs, they were significantly less pronounced compared with those resulting from variations in tillage practices or changes in soil depth.

In addition, the correlation between soil physical properties, soil imaged pore characteristics, and soil saturated water conductivity was analyzed (Table 2). The results indicated that soil PR, FD, anisotropy, and confining layer pore radius were not significantly correlated with soil saturated water conductivity (*p* > 0.05), while soil BD, SMC, and pore specific SA were significantly negatively correlated with soil saturated water conductivity (*p* < 0.01). There was a significant positive correlation with other parameters (*p* < 0.01), and the correlation degree with global connectivity was the highest (r = 0.87∗∗, *p* < 0.01), indicating that pore connectivity played an important role in soil saturated water conductivity.

**Table 2:** The correlation between soil physical properties, soil pore characteristic parameters, and saturated hydraulic conductivity

	BD	PR	SMC	*ϕ*	SA	FD	Γ	CP	DA	HR	MRLL
ln(Ks)	−0.68∗	−0.04	−0.62∗∗	0.80∗∗	−0.72∗∗	0.47	0.87∗∗	0.84∗∗	−0.23	0.74∗∗	0.17

BD, bulk density; PR, pnetration resistance; SMC, soil liquid moisture content; ϕ, porosity; SA, specific surface area; CP, compactness; FD, fractal dimension; DA, degree of anisotropy; Γ, global connectivity; MRLL, mean pore radius of the limiting layer; HR, hydraulic radius.

∗ represents significance at the *p* < 0.05 level, while ∗∗ represents significance at the *p* < 0.01 level.

**Figure 6:**
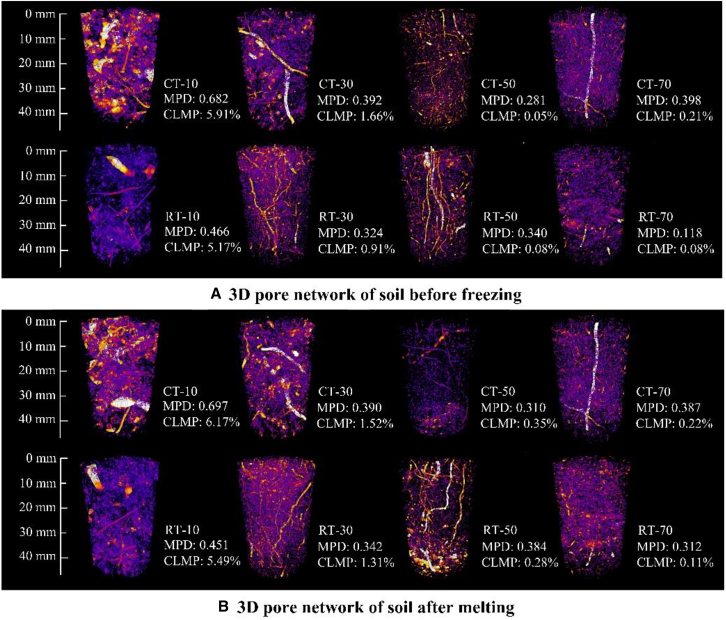
3D thickness map of the imaged pore network under different tillage treatments and soil layers (A) 3D pore network of soil before freezing. CT represents conservation tillage, RT represents rotary tillage, MPD is the mean pore diameter (mm), and CLMP is the connected largest macroporosity. (ROI: diameter 30 mm, height 48 mm). (B) 3D pore network of soil after melting.

According to the 3D images of the selected region of interest (ROI), in the 0–20 cm soil layer, a greater number of irregular macropores were observed under both tillage methods (Figure 6). Additionally, the average diameter of these soil pores was larger, and interconnected macropores comprised a higher proportion of the total imaged pores, resulting in a relatively complex pore structure. In the 20–40 cm soil layer, interconnected macropore channels remained clearly visible under both treatments; however, conservation tillage exhibited more tubular biological pores and larger pore sizes. Conversely, in soil layers exceeding 40 cm in depth, there was a marked reduction in irregular macropores and interconnected pores, with an increasing tendency for pores to be more independent. Simultaneously, as soil depth increased, the maximum connectivity porosity associated with conservation tillage decreased from 6.17% to 0.22%, which may significantly impede subsoil permeability.

Under the influence of FTCs, the maximum porosity of soil connectivity in 0–20 cm soil layer increased under the two tillage treatments, while the maximum porosity of soil connectivity in other soil layers showed different change rules. However, it is worth noting that after FTCs, it was observed visually that some of the originally interconnected macropore channels were broken. This phenomenon indicates that FTCs can lead to displacement within the soil matrix and alter the original network of soil pores.

In order to further investigate the effects of initial soil conditions and freeze-thaw parameters on the variation of soil pore characteristics, several independent variables were selected: soil BD, PR, initial water content of soil layer (IMC), DSF, soil layer location (SL), and average temperature during freeze-thaw period (AT). The variation of soil pore characteristic parameters before and after FTC was used as the dependent variable for multiple stepwise regression analysis. The results indicated that the fitting effect of the variation of imaged porosity, tightness, and the difference of the maximum limiting layer pore radius before and after FTCs was not good; therefore, the results were not shown in the Table 3. The other indicators had good fitting effect and were of significance (Table 3). Among them, DSF provided 85.8%, 88.2%, 84.9%, and 72.7% explanations for ΔSA, ΔFD, ΔDA, and ΔHR, respectively, and BD provided 72.7% explanations for ΔΓ.

**Table 3:** Stepwise multiple regression model between soil initial conditions, freeze-thaw cycling parameters, and changes in soil pore characteristic parameters

Stepwise multiple regression model	*R*^*2*^	*F*	*P*
ΔSA = 0.942∗DSF	0.858	31.328	0.005
ΔFD = 0.952∗DSF	0.882	38.394	0.003
ΔΓ = 0.921∗BD	0.810	22.293	0.009
ΔDA = −0.937∗DSF	0.849	29.008	0.006
ΔHR = −0.884∗DSF	0.727	14.330	0.019

Δ represents the difference between the soil pore characteristic parameter indicators after and before freeze-thaw cycling.

BD, bulk density; DSF, duration of soil freezing; SA, specific surface area; FD, fractal dimension; DA, degree of anisotropy; Γ, global connectivity; HR, hydraulic radius.

### Effects of tillage methods and freeze-thaw cycles on soil nutrients content

The effects of FTCs on the total SOC and soil nutrient levels—including total nitrogen (TN), total phosphorus (TP), total potassium (TK), available nitrogen (AN), available phosphorus (AP), and available potassium (AK)—in the 0–20 and 20–40 cm soil layers under different tillage methods are presented in Table 4. Before soil freezing, in the 0–20 cm soil layer, only TP exhibited a significantly higher concentration under conservation tillage compared with rotary tillage. In the 20–40 cm soil layer, both TN and TP were significantly greater under conservation tillage than under rotary tillage. After soil melting, there was a significant decrease in both TN and AN within the 0–20 cm layer for rotary tillage. Conversely, AN in the 20–40 cm layer showed a significant increase. The FTCs facilitated the decomposition of organic matter and enhanced the release of TN while promoting its transformation into available forms. Furthermore, in the 20–40 cm soil layer under rotary tillage treatment, there was a notable decline in TK; however, slight increases were observed for AN, AP, and AK. This indicates that after undergoing FTCs, nutrients within the soil were leached downward.

**Table 4:** The impact of freeze-thaw cycles on soil nutrient content in the 0–20 cm and 20–40 cm soil layers under different tillage methods

Stage	Treatment	Depth	SOC	TN	TP	TK	AN	AP	AK
		(cm)	(g·kg^−1^)				(mg·kg^−1^)		
Before freezing	CT	0–20	29.2 ± 2.9Aa	0.25 ± 0.01Aa	1.09 ± 0.03Aa	21.1 ± 0.5Aa	230 ± 30.6Aa	25.7 ± 18.5Aa	169 ± 18.1Aa
	RT	0–20	30.4 ± 1.1Aa	0.25 ± 0.00Aa	0.98 ± 0.05Ba	20.9 ± 0.5Aa	220 ± 26.6Aa	42.1 ± 18.3Aa	188 ± 21.3Aa
	CT	20–40	24.0 ± 2.0Aa	0.20 ± 0.01Aa	0.90 ± 0.05Aa	21.0 ± 0.5Aa	238 ± 29.7Aa	14.4 ± 5.9Aa	177 ± 14.2Aa
	RT	20–40	22.6 ± 2.4Aa	0.17 ± 0.01Ba	0.74 ± 0.03Ba	21.1 ± 0.0Aa	235 ± 19.4Ab	19.7 ± 4.7Aa	185 ± 30.1Aa
After melting	CT	0–20	28.2 ± 3.1Aa	0.24 ± 0.01Aa	1.07 ± 0.04Aa	21.0 ± 0.6Aa	211 ± 35.5Aa	30.3 ± 18.2Aa	162 ± 22.0Aa
	RT	0–20	28.3 ± 1.3Aa	0.23 ± 0.01Ab	0.93 ± 0.06Ba	20.7 ± 0.6Aa	159 ± 21.0Ab	51.3 ± 27.6Aa	154 ± 18.5Aa
	CT	20–40	23.5 ± 2.1Aa	0.21 ± 0.01Aa	0.89 ± 0.05Aa	20.9 ± 0.6Aa	261 ± 36.2Aa	15.4 ± 7.6Aa	180 ± 16.3Aa
	RT	20–40	21.8 ± 2.6Aa	0.18 ± 0.01Aa	0.73 ± 0.03Ba	21.0 ± 0.6Ab	281 ± 28.6Aa	22.4 ± 6.3Aa	177 ± 35.9Aa

CT, conservation tillage; RT, rotary tillage. SOC, soil organic carbon; TN, total nitrogen; TP, total potassium; TK, total phosphorus; AN, alkaline nitrogen; AP, available phosphorus; AK, available potassium.

Data are represented as mean ± SEM.

Capital letters indicate significant differences between the two tillage methods, whereas lowercase letters denote significant differences within the same treatment before freezing and after thawing.

**Figure 7:**
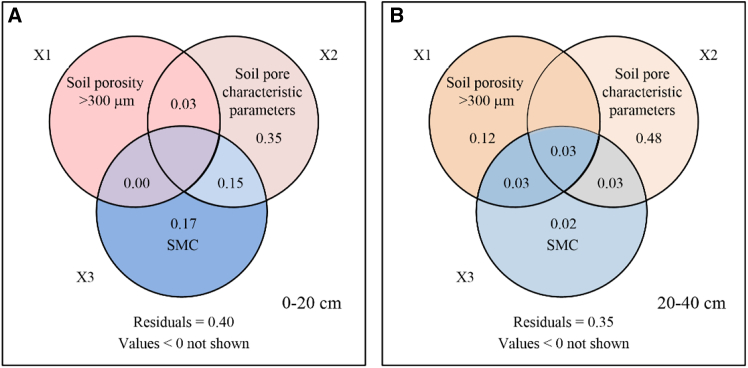
The impact of soil moisture and pore characteristic parameters across various soil layers on soil nutrient availability (A) Soil moisture and pore traits affect nutrient availability in the 0–20 cm layer. (B) Soil moisture and pore traits affect nutrient availability in the 20–40 cm layer.

To further investigate the contribution of soil pores to changes in soil nutrients, the variable portioning analysis (VPA) was employed to assess the impact of various soil pore characteristics on nutrient levels within the 0–20 cm and 20–40 cm soil layers (Figure 7). The indicators for soil nutrients included SOC, TN, TP, TK, AN, AP, and AK. Based on the data presented in Tables 2 and 3, SA, HR, and Γ were selected as environmental factor 1; macroporosity >300 μm was designated as environmental factor 2; and SMC was identified as environmental factor 3. The results are illustrated in Figure 7. For the 0–20 cm soil layer, it was observed that macroporosity >300 μm did not contribute to the overall variation in soil nutrients. The contribution rate of the selected soil pore characteristic parameters to this total variation accounted for 35%, while the contribution from SMC represented an additional 17%. In contrast, for the deeper layer of 20–40 cm, the respective contribution rates of these three environmental factors to total nutrient variation were recorded at 12%, 48%, and a mere 2%.

## Discussion

### Effects of different tillage methods on soil pore structure

Under various tillage methods, the differences in the quality, power, and operational depth of agricultural machinery and tools lead to distinct patterns in soil PR and BD along the vertical profile.^23^ This study demonstrated that the PR under conservation tillage within the 0–30 cm soil layer was higher than that observed under rotary tillage. Similarly, for the 40–60 cm soil layer, PR exceeded that of rotary tillage; however, the difference between these two layers was not significant. Notably, from a vertical perspective, protective tillage treatment resulted in relatively elevated PR at the 20 cm soil layer (Figure 2C). Although only one distinct compacted layer was observed at the 20 cm depth in this study, this pattern is broadly consistent with the findings of Wang et al.,^24^ who reported that long-term mechanical operations can lead to the formation of three vertically alternating hard layers. The less pronounced expression of additional compacted layers may be attributable to the relatively short duration of conservation tillage (two years) in the present experiment. This confirms that under protective tillage conditions, there is increased PR in the topsoil layer while exhibiting minimal compaction effects on deeper layers.

A traditional perspective is that rotary tillage can alleviate surface soil compaction but easily lead to soil ecological degradation,^25^ while conservation tillage (no tillage or less tillage) has been shown to enhance the inherent water retention capacity and hydraulic conductivity through the accumulation of organic matter and biological pores.^26^ In this study, we found that the total imaged porosity within the 0–20 cm soil layer was comparable between both tillage methods. However, under conservation tillage treatment, a greater proportion of pores exceeded 500 μm in diameter compared with those measuring between 200 μm and 400 μm under rotary tillage treatment (Figure 3A). These large pores, exceeding 500 μm in diameter, facilitate rapid water movement, promote adaptive root growth, and encourage crop roots to preferentially extend into areas with low mechanical resistance.^27^ Additionally, they contribute to the stability of soil structure.^28^ This observation is further supported by the three-dimensional thickness map of the imaged pore network (Figure 6) as well as characteristics of soil biological pores described by Zhang et al.,^18^ which confirm from a morphological standpoint that conservation tillage fosters more circular tubular biological pores and an increased number of interconnected macropores. Additionally, Figure 4 illustrates that within the topsoil layer (0–20 cm), imaged porosity was greater under conservation tillage treatment while rotary tillage exhibited a higher quantity of individual pores. This phenomenon corroborates our findings that pore sizes tend to be larger with more concentrated distributions under conservation tillage practices. Similarly, at depths ranging from 0 to 20 cm, conservation tillage demonstrated superior water transport capabilities compared to rotary tillage treatments (Table 1), likely due to enhanced root systems facilitating stable preferential flow paths in the soil.^29^ The aforementioned findings align with those of Chakraborty et al.,^30^ indicating that the implementation of conservation tillage yields consistent results in short-term tests. However, as soil depth increases, the imaged porosity of the soil decreases progressively across layers, with a significant reduction observed in porosity greater than 500 μm. Additionally, both the imaged porosity and the number of soil pores exhibit gradual fluctuations corresponding to changes in depth, which is also consistent with the observations made by Luo et al.^12^

Compared with the rotary tillage treatment, the conservation tillage treatment had lower specific SA, FD, compactness, anisotropy, and higher global connectivity, HR, and pore radius of the restricted layer in the 0–20 cm soil layer. Based on the definitions of each indicator as introduced by those findings,^13^^,^^22^ it is confirmed that under conservation tillage conditions, pore shapes are more regular and soil cracks occur more frequently when rotary tillage is subjected to potential external disturbances during operation.^31^ This study further established that both HR and global connectivity serve as reliable predictors for actual hydraulic conductivity in topsoil (Table 3). In relation to deeper soil layers (60–80 cm), although conservation tillage resulted in larger connected pores and an increased pore diameter within the maximum confining layer (Figure 5), enhanced imaged porosity did not necessarily correlate with improved hydraulic conductivity. Therefore, evaluating soil hydraulic conductivity solely based on global connectivity or average pore radius of the maximum confining layer may lead to inaccuracies.

### Effect of freeze-thaw cycles on soil pore structure

Previous studies have confirmed that FTCs can reduce soil compaction and affect soil hydraulic properties.^32^^,^^33^ In this study, following natural FTCs, soil PR decreased at various depths, with the exception of the 0–20 cm soil layer treated by rotary tillage. However, considering that soil PR was greatly affected by SMC.^34^ In addition, water migrates continuously with the freezing front during the FTCs.^35^ In the case of SMC difference, PR alone could not accurately determine whether soil compaction was improved. Combined with the changes of soil BD in this study (Figure 2), it can be found that the total porosity of the soil in this study increased slightly under the influence of natural FTCs. Therefore, we conclude that the FTCs examined in this study contributed to a reduction in the state of soil compaction.

However, the influence of FTCs on soil porosity does not consistently lead to an increase. One study conducted an indoor freeze-thaw simulation test using soil columns with low BD, revealing that FTCs in uncompacted soil can promote soil melt settlement and decrease the imaged porosity of the soil.^36^ Another study also observed that FTCs reduced both macroporosity and specific SA of the soil.^20^ By comparing the experimental conditions from these studies with those of this research, and in conjunction with the patterns illustrated in Figure 3, we indicated that ice lenses preferentially form in macropores during the phase transition of soil water. When soil pores are excessively small (less than 50 μm), capillary pores become obstructed due to strong capillary forces and water migration, leading to insufficient space for the growth of ice crystals.^37^ It can be concluded that pores larger than 300 μm are more susceptible to FTCs under natural conditions. Meanwhile, the ice crystals formed during the freezing process exert pressure on soil particles, potentially leading to the expansion of biological pores created by root systems and earthworm channels.^38^ During the melting stage, soil animal activities resume, resulting in the re-excavation and expansion of these pores. This cyclical change influences both the dynamic distribution and functionality of biological pores.^39^ The 0–20 cm layer of soil, characterized by a higher abundance of biological pores, will further enhance pore connectivity following FTCs, thereby improving hydraulic conductivity as depicted in Figure 5. According to the results of stepwise regression analysis, after screening and excluding collinear factors, only DSF can explain the variation of some soil characteristic parameters among many freeze-thaw factors. Under the influence of FTCs, DSF and its related indicators can intervene in the recombination of soil pores, thus increasing the structural complexity of the upper soil and making soil pore morphology more regular.^31^ The aforementioned analysis also confirmed that FTCs can change the soil pore structure. Nonetheless, it is important to note that when comprehensively comparing the effects of both FTCs and tillage on soil porosity, tillage exerts a more pronounced effect on porosity than do FTCs processes.

Tillage methods, such as deep tillage, can directly generate new pores exceeding 500 μm through mechanical fragmentation. However, prolonged deep plowing heightens the risk of soil erosion.^3^ Compared with the findings of Ding et al.^11^ regarding long-term conservation tillage practices in non-freeze-thaw regions, conservation tillage significantly minimizes soil disturbance, promotes the accumulation of soil organic matter, and increases the prevalence of large pores within the 0–10 cm soil layer. This research also demonstrated the advantage of conservation tillage in alleviating soil compaction.^11^ Based on the findings of this study, conservation tillage combined with FTCs significantly enhanced the development of soil macropores exceeding 300 μm in diameter. This interaction established a positive feedback loop. The biological pores generated by conservation tillage were more prone to forming ice crystals during phase transitions. The FTCs further enlarged soil pores and mitigated soil compaction. During the melting phase, nutrients within the soil could be more effectively transported through these expanded pores. Consequently, integrating conservation tillage with the management of soil freeze-thaw processes is anticipated to emerge as an efficient and environmentally sustainable strategy for alleviating soil compaction in agricultural fields in the future.

### The influence of soil pores on soil nutrients

Soil pores constitute a crucial element of soil structure, with their size, distribution, and connectivity exerting direct influence on the movement of water and nutrients within the soil.^39^ Generally, larger soil pores can significantly impact regional hydrological processes due to their enhanced capacity for soil water infiltration.^40^ In contrast, smaller soil pores not only markedly improve the soil’s water retention capabilities^41^ but also augment nutrient adsorption by increasing the specific SA of the soil.^42^ Furthermore, they can affect the decomposition process of soil carbon by modifying micro-environmental conditions within the soil.^43^ In this study, tillage methods and FTCs were two main factors affecting soil pore structure. Moreover, under the influence of FTCs, TN and AN in the 0–20 cm soil layer under rotary tillage treatment significantly decreased, while there was no significant change in soil nutrients under conservation tillage treatment. This may be related to the stability of soil pore structure under conservation tillage (Figures 5B and 5D). Meanwhile, VPA data show that soil nutrients in 0–20 cm are not affected by pores >300 μm, indicating that within the threshold range of soil reaching a fixed porosity, SMC and the aeration and water permeability of the soil are the main factors influencing nutrient distribution. In the 20–40 cm soil layer, close to the plow layer, the contribution rates of soil pore characteristic parameters, large porosity, and SMC to nutrient variation were 12%, 48%, and 2%, respectively. The number of pores >300 μm in the soil and the sudden drop in soil water conduction capacity (Figure 5A; Table 1). SMC plays an important role in the process of transporting soil nutrients. The relatively dense pore structure avoids nutrient loss during the downward leaching process of nutrients (Table 4).

Therefore, the influence of soil pore characteristics on nutrient availability varies with both soil layer depth and tillage practices. In the topsoil, smaller pores and liquid moisture content are the primary factors governing nutrient distribution. Conversely, in deeper soil layers, the impact of high porosity on nutrient variability becomes more pronounced. The FTCs further influence the decomposition, transformation, and leaching of nutrients by modifying the soil’s pore structure. Conservation tillage plays a crucial role in reducing nutrient leaching and enhancing soil fertility by preserving the stability of the soil pore architecture.

### Limitations of the study

Although this study elucidated the influence of soil pore characteristics on nutrient dynamics under FTCs and clarified the role of pore structure in mediating nutrient distribution across different tillage practices, several limitations warrant acknowledgment.

First, the X-ray CT measurements are subject to inherent technical constraints. The spatial resolution of 60 μm employed in this study precludes reliable detection of fine pores—particularly micropores and smaller mesopores—that significantly contribute to water retention and serve as critical habitats for soil microorganisms. Furthermore, pore segmentation during image processing relied on a single global intensity threshold; the selection of this threshold inevitably involves operator-dependent judgment, potentially compromising the absolute accuracy and reproducibility of the derived pore metrics. Critically, current X-ray CT methodology cannot reliably differentiate biologically formed pores (e.g., earthworm burrows and root channels) from those generated by physical processes (e.g., freeze-thaw-induced sedimentation or mechanical disturbance from tillage implements). This limitation hinders the mechanistic understanding of how pore origin governs functional stability—such as preferential flow pathways, gas diffusivity, and the provision of microbial niches.

Second, the experimental design and statistical power impose additional constraints. The study was conducted at a single site characterized by a specific black soil type and local climate regime; thus, the observed quantitative relationships may not be directly generalizable to other pedoclimatic contexts. Moreover, logistical and cost limitations constrained both the number of soil cores subjected to X-ray CT scanning and the extent of field sampling, resulting in relatively low statistical power. Although major treatment effects were statistically discernible, more subtle yet ecologically relevant responses—such as shifts in nutrient speciation kinetics or microscale biogeochemical hotspots—may have remained undetected. Consequently, the robustness of the conclusions under conditions of greater environmental or management-induced variability remains to be validated.

Third, and most critically, the observed linkage between pore characteristics and nutrient status is correlational rather than causal. This study did not assess the composition, diversity, or functional activity of soil microbial communities—including bacteria, fungi, and key extracellular enzymes such as urease, dehydrogenase, and cellulase—nor did it quantify root architectural traits (e.g., length, density, and branching) or their dynamic interactions with the soil pore network. Microorganisms and plant roots are the principal biological drivers of biogeochemical cycling for carbon, nitrogen, and phosphorus; their metabolic activity is highly sensitive to alterations in pore structure and to water-phase transitions induced by FTCs. In the absence of measurements for these critical mediating variables, the present dataset cannot elucidate the underlying mechanistic pathways by which pore modifications govern the observed nutrient dynamics. Future work must, therefore, integrate targeted microbial assays, high-resolution root-system phenotyping, and advanced causal inference approaches—such as structural equation modeling—to move beyond correlation and establish a mechanistic understanding of pore-nutrient relationships.

## Resource availability

### Lead contact

Requests for further information and resources should be directed to and will be fulfilled by the lead contact, Qinglin Li (qlli@neau.edu.cn).

### Materials availability

This study did not generate new unique materials.

### Data and code availability

• All data supporting findings of this study are provided within the article.
• This paper does not report original code.
• Any additional information required to reanalyze the data reported in this article is available from the lead contact upon request.

## Acknowledgments

This research was supported by 10.13039/501100012166National Key Research and Development Program of China (2022YFD1500905–4), 10.13039/501100001809National Natural Science Foundation of China (52422902 and 52409075), Program for Young Talents of Basic Research in Universities of Heilongjiang Province (YQJH2025018), and 10.13039/501100005046Natural Science Foundation of Heilongjiang Province of China (QC2025E001).

## Author contributions

Q.L., R.H., and Z.Z. designed the project; Q.L. collected and analyzed the data; Q.L., R.H., Z.Z., Q.F., and T.L. wrote the paper.

## Declaration of interests

The authors declare no competing interests.

## STAR★Methods

### Key resources table

**Table:** 

REAGENT or RESOURCE	SOURCE	IDENTIFIER
**Software and algorithms**		

ImageJ	Starkloff, T., Larsbo, M., Stolte, J., Hessel, R., Ritsema, C., 2017. Quantifying the impact of a succession of freezing-thawing cycles on the pore network of a silty clay loam and a loamy sand topsoil using X-ray tomography. Catena 156, 365–374.^20^	https://imagej.nih.gov/ij/
SPSS Statistics 26	IBM	https://www.ibm.com/cn-zh/products/spss-statistics
Origin 2024	Originlab	https://www.originlab.com/

**Other**		

Phoenix V|tome|x M300	Waygate Technologies	https://www.bakerhughes.com/
SC900	Spectrum Technologies	https://www.osmre.gov/sites/default/files/inline-files/Soil-Compaction-Meter-Spectrum-Technologies-SC-900-MCR-2025_0.pdf
TDR-315H	Acclima	https://acclima.com/tdr-soil-moisture-sensor-user-manual/

### Method details

#### Overview of the study area

The experimental site for this study was located in Hailun City, Heilongjiang Province, Northeast China (126.79E, 47.43N). The soil formation in this area began with loess deposited under the natural meadow of the Quaternary, containing 14.23% sand, 59.39% silt and 26.38% clay. According to the American soil diagnostic classification system, this soil is categorized as Mollisols. In this region, summers are characterized by hot and rainy conditions while winters are cold and dry. The average annual temperature is about 1.5°C, the average annual radiation is about 113 MJ cm^−2^, the average annual precipitation is about 530 mm, the winter precipitation is snow, winter and spring accompanied by seasonal freeze-thaw phenomenon, belongs to the temperate continental monsoon climate. The basic physical and chemical properties of the topsoil were determined before the test began as follows: The pH value of the soil was 6.79, the content of alkali-hydrolytic nitrogen was 206.72 mg kg^−1^, the content of available phosphorus was 50.41 mg kg^−1^, the content of available potassium was 159.13 mg kg^−1^, the content of total potassium was 17.29 g kg^−1^, the content of total phosphorus was 0.74 g kg^−1^, the total nitrogen content was 1.83 g kg^−1^, the organic matter content was 67.62 g kg^−1^, and the cation exchange capacity was 33.17 cmol kg^−1^.

#### Experimental design and field management

The experimental site was established and put into use in 2022, employing a maize continuous cropping tillage system. In this study, two treatments were set up: (1) Conservation tillage treatment (CT), which involved no-tillage throughout the year, utilizing a no-till seeder to simultaneously complete sowing and suppression operations; (2) Rotary tillage straw return treatment (RT), where maize straw was returned to the field after each annual harvest, with an operational depth of 15–18 cm. Each treatment was repeated three times, with a total of 6 plots, each with an area of 40 m^2^ (10 m × 4 m), and the management measures such as fertilization and pesticide use within each plot were consistent. At the same time, in order to further explore the temperature changes of soil at different depths during the freeze-thaw period, soil temperature sensors were embedded in the soil layers of 10 cm, 30 cm, 50 cm and 70 cm in each test plot before the start of soil freezing (TDR-315H, Acclima, USA). Based on the soil temperature records (Figure 1), the surface soil in the study area experienced approximately 12–15 complete freeze-thaw cycles (daily minimum temperature below 0°C followed by above 0°C) during the monitoring period from November 2023 to March 2024.

#### Sample collection and analysis

Soil samples were collected following maize harvest in October 2023 and prior to the commencement of spring ploughing in April 2024. The sample collection methods employed were as follows: PVC columns (diameter 6.0 cm, height 10.0 cm) were utilized to extract undisturbed soil layers at depths of 5–15 cm, 25–35 cm, 45–55 cm, and 65–75 cm from plots subjected to two different treatments—conservation tillage and rotary tillage—for X-ray CT scanning. After the initial scans, the original samples were returned to their respective soil layers in the field. Subsequently, PVC soil samples were extracted again for CT scanning before spring ploughing commenced. To minimize soil disturbance and prevent water loss during transportation, the PVC soil columns were wrapped in plastic and placed within shock-proof bags. In addition, ring knives (volume of 100 cm^3^) were used to collect undisturbed soil samples from various layers under differing treatments. Soil bulk density (BD) was determined by drying method, and the soil saturated water conductivity (Ks) was determined by constant head method. Soil penetration resistance (PR) was measured using a soil compactness meter (SC900, Spectrum, USA).

#### X-ray computer tomography scanning and image analysis techniques

The sample soil columns were scanned using an industrial X-ray computed tomography scanner (Phoenix v | tome | x m 300, GE, Sensing and Inspection Technologies, GmbH, Wunstorf, Germany). The scanning pixel resolution was set to 60 μm, with a scanning voltage of 180 kV and a current of 160 μA. The exposure time for each scan was 334 mS. During the scanning process, each soil column was rotated at a constant speed of 360° on the sample table, and about 1700 two-dimensional slice images were collected during this process. For the scanned original images, Phoenix Datos x software was used to reconstruct the images, and then VG StudioMAX 3.5 software was used to export 16-bit Tiff grayscale images.

Each sample image was processed using ImageJ software (https://imagej.nih.gov/ij/). Through batch operations, the image formats were adjusted to 8-bit. The “Brightness & Contrast” function in the software ribbon was utilized to modify the image contrast to a moderate level of brightness and darkness, enabling effective differentiation between soil pores and the soil matrix. To mitigate edge effects in the images and eliminate artifacts present in the original images, as well as interference caused by soil vibration during sampling, a soil core with a central diameter of 30 mm and a height of 45 mm was selected as the region of interest (ROI). The “Median” filtering method within the software was employed to reduce image noise, with a radius setting of 2.0. Subsequently, the “Threshold” function was applied; by default, the automatic threshold method implemented in ImageJ (Maximum entropy‘s algorithm) was applied to binarize the images, and the results were manually checked for consistency, ultimately resulting in an image depicting soil pore distribution. After completing the above steps, the ImageJ software was used to calculate the imaged porosity (*ϕ*), specific surface area (SA), compacting degree (CP), hydraulic radius (HR), fractal dimension (FD), degree of anisotropy (DA), global connectivity (Γ), and maximum limiting layer pore radius (MRLL) of the soil ROI region, respectively. Furthermore, the local thickness algorithm in Bone J uses the maximum total inscribed sphere to calculate thickness. With this feature, we can obtain different soil pore size distributions (60–200 μm, 200–300 μm, 300–400 μm, 400–500 μm, >500 μm). Among them, ‘Imaged porosity’ refers to the porosity derived from computer tomography image segmentation, which mainly represents pores >60 μm due to the resolution limit. And ‘Porosity’ refers to the degree of porosity calculated based on the soil bulk density.

### Quantification and statistical analysis

#### Statistical analysis

This investigation employed IBM SPSS Statistics 26 and Origin 2024 software for processing, plotting, and tabulating experimental data. Independent samples *t* test were employed to investigate the differences in saturated water conductivity of the same soil layer before freezing and after thawing under identical treatment conditions, as well as to assess the variations in saturated water conductivity across different treatments applied to the same soil layer. The significance level (α) was established at 0.05, and a two-tailed test was conducted. Pearson correlation analysis was utilized to evaluate the relationships among soil physical properties, soil pore characteristic parameters, and saturated water conductivity. Additionally, multiple linear regression equations were implemented to quantify the extent of influence exerted by initial soil conditions and freeze-thaw parameters on variations in soil pore characteristic parameters using a stepwise screening method.
